# The assessment of the clinical usefulness of suicide-specific syndrome inventories – a retrospective study with psychiatric inpatients

**DOI:** 10.1192/j.eurpsy.2023.1065

**Published:** 2023-07-19

**Authors:** V. Voros, S. Fekete, C. Molnar, J. Szabo, T. Tenyi, P. Osvath

**Affiliations:** University of Pecs, Pecs, Hungary

## Abstract

**Introduction:**

There is growing evidence on the existence and the clinical usefulness of the recently described Suicide-Specific Syndromes (SSS), such as the *Acute Suicidal Affective Disturbance (ASAD)* and the *Suicidal Crisis Syndrome (SCS).* Many researchers and clinicians agree that Suicide-Specific Syndromes, as a distinct diagnostic category should be included in the major diagnostic and classification systems, such as the Diagnostic and Statistical Manual of Mental Disorder (DSM) or the International Classification of Diseases (ICD). In addition to their novelty in symptomatic and nosologic classification, the recently developed *Acute Suicidal Affective Disturbance Inventory (ASADI)* and the *Suicidal Crisis Inventory (SCI)* also provide an opportunity to objectively measure the current suicidal emotional and mental state by validated tools.

**Objectives:**

To assess the clinical usefulness of the ASADI and the SCI compared to each other and to traditional suicide risk assessment tools based on classical suicide risk factors.

**Methods:**

A self-administered questionnaire battery and a semi-structured interview were completed with 100 psychiatric inpatients consecutively treated with depressive disorders and/or suicidal behaviour in a university clinic in 2021. Besides the ASADI and the SCI, the self-administered battery included depression screening tools, such as the Beck Depression Inventory - Short Form (BDI-SF) and the Patient Health Questionnaire - 9 items (PHQ-9). Traditional suicide risk factors were assessed by clinical judgement and with the Brief Suicide Questionnaire (BSQ).

**Results:**

According to our preliminary results, the ASADI and the SCI recognize Suicide-Specific Syndromes as distinct diagnostic entities. Furthermore, ASADI and SCI detect suicidal behaviour as effectively as traditional suicide risk assessment tools, and may be more effective in assessing imminent suicide risk. There were no significant differences in detecting suicidal behaviour and in assessing suicide risk between the ASADI and the SCI.

**Conclusions:**

Suicide-Specific Syndromes (ASAD, SCS) use well-defined diagnostic criteria for suicidal behaviour. The recently developed different tools for assessing Suicide-Specific Syndromes, such as the ASADI and the SCI may be helpful tools for the clinicians to assess suicidal behaviour and imminent suicide risk in their clinical practice.

**Disclosure of Interest:**

None Declared

